# Malignant pulmonary embolism associated with renal sarcoma: A case report

**DOI:** 10.1097/MD.0000000000019943

**Published:** 2020-05-08

**Authors:** Jun Ho Yang, Dae Hyun Song, Chunwoo Lee, Dong Hoon Kang, Jae Jun Jung, Sung Hwan Kim, Joung Hun Byun, Jong Woo Kim, Seong Ho Moon

**Affiliations:** a Department of Thoracic and Cardiovascular Surgery, Changwon; b Gyeongsang National University School of Medicine; c Gyeongsang Institute of Health Science, Jinju; d Department of Pathology, Gyeongsang National University Changwon Hospital; e Department of Urology, Gyeongsang National University School of Medicine and Gyeongsang National University Changwon Hospital, Changwon, Republic of Korea.

**Keywords:** pulmonary embolectomy, pulmonary tumor embolism, radical nephrectomy, renal sarcoma

## Abstract

**Rationale:**

The preoperative diagnosis of massive pulmonary tumor embolism associated with renal neoplasms is relatively rare. In most cases, pulmonary tumor embolism is detected intraoperatively during renal tumor resection. Moreover, primary renal sarcoma is rare, and primary renal sarcoma complicated by pulmonary tumor embolism is extremely rare; accordingly, there is no optimal treatment for such cases. Herein, we report a case of renal sarcoma associated with pulmonary tumor embolism.

**Patient concerns:**

A 39-year-old man was admitted to the emergency room owing to the sudden onset of dyspnea and palpitation.

**Diagnosis:**

Contrast-enhanced computed tomography (CT) revealed a large mass in the right kidney involving the infrahepatic inferior vena cava, with massive pulmonary emboli in both the pulmonary arteries.

**Interventions:**

Emergency pulmonary embolectomy with radical nephrectomy was performed.

**Outcomes:**

The patient experienced apparent remission of dyspnea, and resolution of right ventricle dysfunction. However, although remnant emboli were detected in the segmental arteries on postoperative CT, complete resolution of pulmonary embolism was observed after adjuvant chemotherapy.

**Lessons:**

Thus, concomitant cytoreductive nephrectomy with pulmonary embolectomy along with chemotherapy may be effective for patients with renal sarcoma with pulmonary tumor embolism.

## 1 Introduction

Soft tissue sarcoma is a rare malignant tumor of mesenchymal origin, with an incidence of 2 to 3 cases per 100,000 people, contributing to <1% of all adult malignancies.^[1]^ Adult renal sarcomas comprise only 1% of all renal malignancies.^[2,3]^ Owing to the rarity of primary renal sarcoma, the optimal treatment approach is not yet well established.^[2,3]^ Metastatic sarcomas that originate from other sites are usually treated with chemotherapy. In contrast, metastatic renal cell carcinomas (RCCs) show better prognosis after treatment with cytoreductive nephrectomy.^[3]^ However, the effectiveness of resection is not known for renal sarcoma complicated with pulmonary tumor embolism. Therefore, herein, we report a case of renal sarcoma complicated with pulmonary tumor embolism that was successfully treated with cytoreductive nephrectomy with pulmonary embolectomy; we believe that the findings of the current case provide the basis for treatment of such complicated renal sarcomas in future.

## 2 Case report

**Figure 1:**
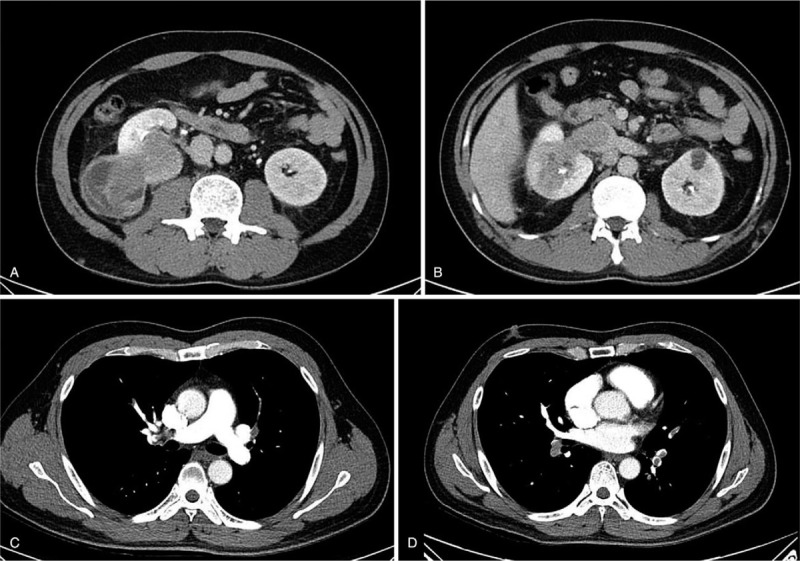
(a) Preoperative abdominal computed tomography scans showing a right renal mass (b) protruding into the inferior vena cava . (c) Preoperative chest computed tomography scans showing pulmonary tumor emboli in the right main pulmonary artery (d) involving both the pulmonary arteries.

**Figure 2:**
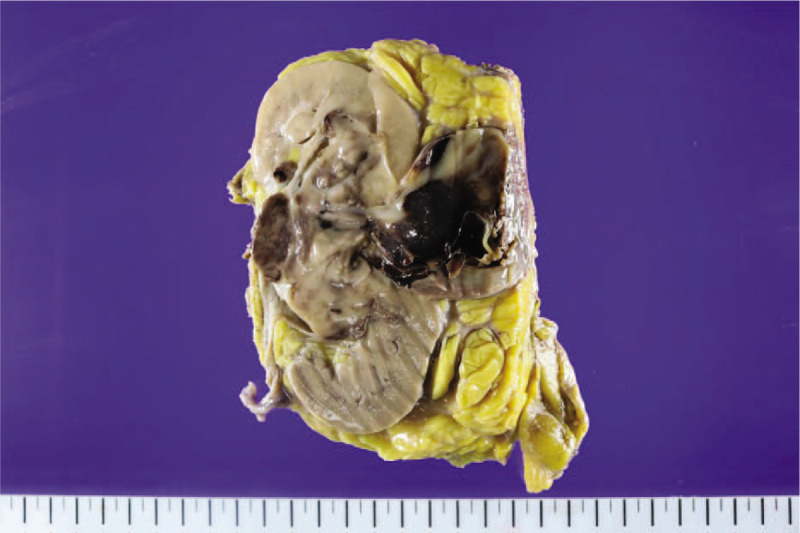
Gross specimen of the tumor in the right kidney obtained via nephrectomy showed a 6-cm mass with a relatively well-defined border. The mass was brown, showing hemorrhage but no necrosis.

**Figure 3:**
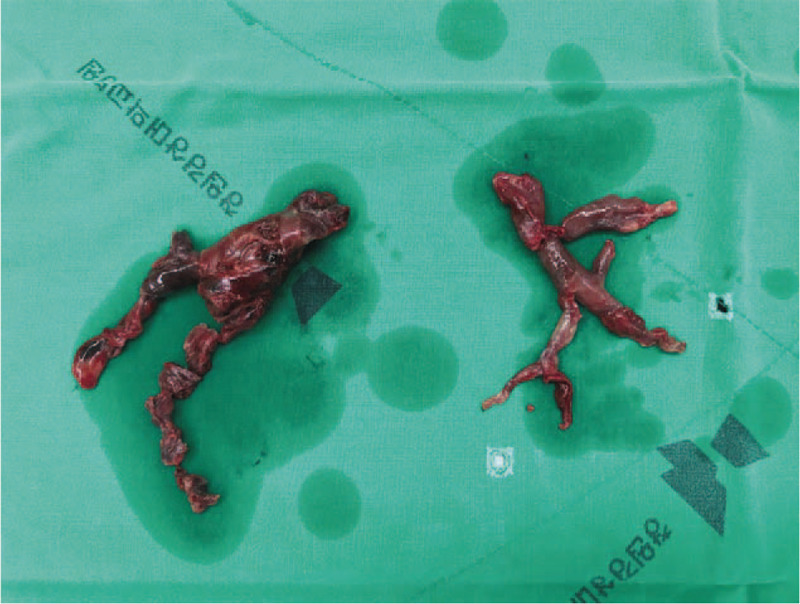
Gross specimen of the emboli from both the pulmonary arteries.

A 39-year-old man with no history of illnesses or medication complained of sudden onset of dyspnea and palpitation and was brought to the emergency room. He had right flank pain the day before, but no hematuria. In addition, he experienced cold sweats and substernal pain, and his body temperature was 37.3°C. On initial electrocardiography, we observed sinus tachycardia (104 beats per minute). The patient's respiratory rate was 23 breaths per minute, with severe hypoxemia, on arterial blood gas analysis (pH, 7.35; pCO_2_, 31 mm Hg; pO_2_, 39 mm Hg; bicarbonate, 17 mmol/L; base excess, 9.0; O_2_ saturation, 75.2%). Contrast-enhanced computed tomography (CT) revealed a 6-cm right renal mass protruding into the infrahepatic inferior vena cava (IVC; Fig. 1a and b) as well as massive pulmonary embolisms in the right main pulmonary artery and both the lobar and segmental arteries (Fig. 1c and d). Transthoracic echocardiography revealed the D-shape of the left ventricle with dilated and decreased contractility of the right ventricle. Considering these findings, we suspected RCC with extension of the tumor thrombus into the IVC and pulmonary tumor embolism; accordingly, in cooperation with urologists, we decided to perform immediate concomitant pulmonary embolectomy with radical nephrectomy. Midline laparotomy was performed by making a median sternotomy incision. After dissection of the right kidney, nephrectomy with longitudinal IVC venotomy was performed for removal of the IVC tumor thrombus (Fig. 2). As the IVC did not show tumor invasion, direct suture repair was performed, as is usually done. Cardiopulmonary bypass was performed via ascending aorta cannulation and bicaval venous cannulation. After cross-clamping the aorta, cardiac arrest was induced. Incisions on both the main pulmonary arteries were made separately, and embolectomy was performed under deep hypothermic total circulatory arrest owing to excessive blood return from the pulmonary arteries (Fig. 3). After the embolectomy was completed, the pulmonary arteries were repaired, the aorta cross-clamp was released, and cardiopulmonary bypass was weaned smoothly. The aorta cross-clamping time was 45 minutes, and the total circulatory arrest time was 3 minutes. Ventilator weaning and extubation were possible on the day of the surgery. The patient was transferred to the general ward the next day On immunohistochemical analysis, the tumor was focally positive for CD10, positive for vimentin, and negative for CD31, CD34, cytokeratin, desmin, HMB45, Melan-A, S100, and SMA. The staining for CD99 was equivocal, and the Ki67 index was 50%. On molecular analysis, the *SYT/SS18* gene study exhibited negativity for translocation and *EWS/FLI1* fusion was not detected. On the basis of all these findings, the pathological diagnosis was an undifferentiated/unclassified sarcoma of the kidneys (grade 2/3).

**Figure 4:**
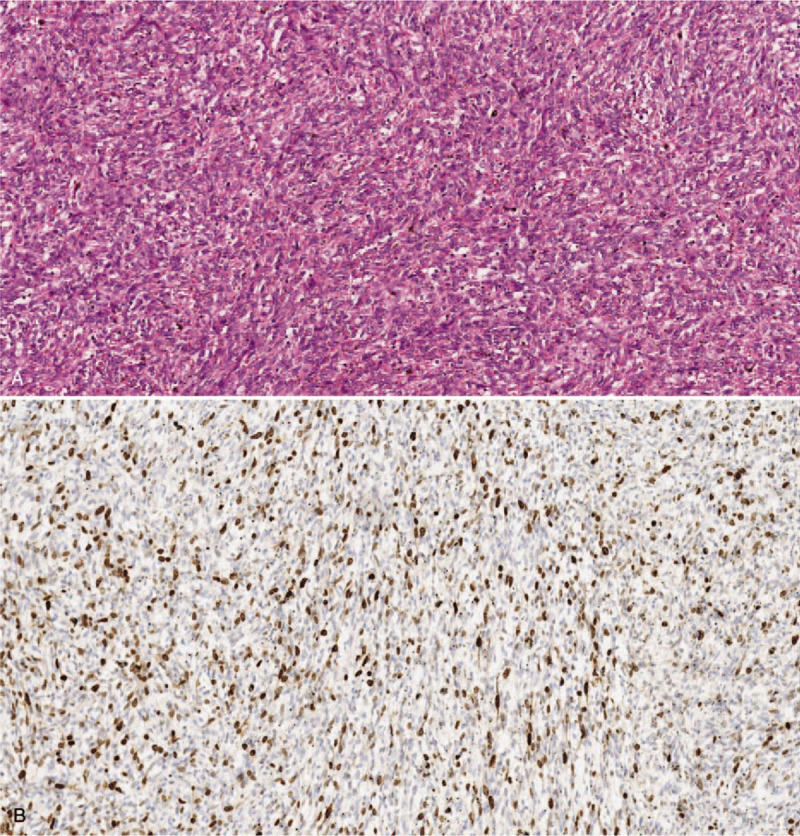
(a) Histopathology findings of the renal tumor (hematoxylin and eosin staining; magnification, 100×); (b) Ki67 immunohistochemical staining (magnification, 100×). The spindle cells with hyperchromatic nuclei showed high cellular density.

**Figure 5:**
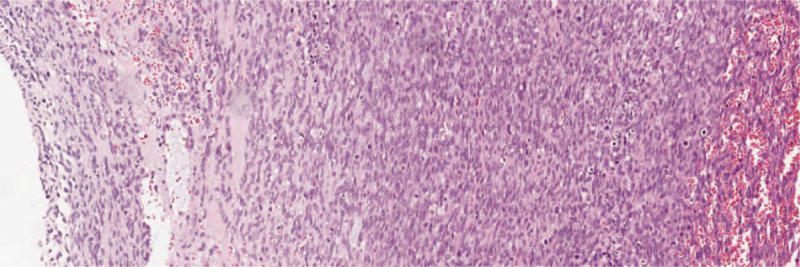
Pulmonary embolus (hematoxylin and eosin staining; magnification, 100×): the spindle cells with hyperchromatic nuclei showed high cellular density.

The tumor involved the perirenal fat tissue and was confined within the Gerota fascia (Fig. 4). The pulmonary emboli were confirmed to be sarcoma emboli (Fig. 5). Postoperative chest CT revealed some remnant emboli in the segmental arteries. The patient was discharged on the ninth operative day without any early complications, and was recommended to undergo follow-up positron emission tomography-computed tomography (PET/CT) and adjuvant chemotherapy. However, for personal reasons, the patient underwent PET/CT and adjuvant chemotherapy at another institute. The patient underwent a course of chemotherapy with doxorubicin and dacarbazine 2 months after surgery. At 8 postoperative months, we were notified that PET/CT revealed no potential dissemination of the malignancy, and the tumor thrombi were completely resolved after chemotherapy. The patient is alive without recurrence 1 year after surgery. The patient provided informed consent for publication of the case.

## 3 Discussion

We report our experience of treating a case of primary renal sarcoma with massive pulmonary tumor embolism, which is a combination of 2 very rare entities. Primary renal sarcoma in adults account for only 1% of all renal cancers,^[2,3]^ and renal neoplasms with massive pulmonary tumor embolism have been reported in limited case series. We hypothesized that we would be 1 step closer to determining the optimal treatment for primary renal sarcoma with massive pulmonary tumor embolism by reviewing each entity separately and then integrating the outcomes of the 2 entities. Accordingly, we reviewed the literature about the treatment of renal sarcoma and pulmonary tumor embolism considering the varying histology of renal tumors, with most cases of RCC, as it is the most common histologic type.

Because of the rarity of primary renal sarcoma in adults, the optimal treatment has not yet been determined; management is mostly based on the treatment outcomes of either sarcoma from other anatomical sites or renal tumors of different histology.^[3]^

Pulmonary tumor embolism in RCC patients is mostly identified intraoperatively during mobilization and removal of the primary tumor.^[4]^ Cases of preoperative diagnosis of pulmonary tumor embolism in RCC are limited in literature, with no reported cases of pulmonary tumor embolism associated with renal sarcoma.^[4]^

A total of 4% to 10% of patients with RCC present with venous tumor thrombus. The presence of a vena cava tumor thrombus increases the patient's risk of distal embolism.^[5]^ Accordingly, RCC with a pulmonary tumor embolism seems to be similar to RCC with IVC thrombus. Therefore, the characteristics of RCC with IVC involvement may indirectly indicate the clinical nature of RCC with pulmonary tumor embolism. Accordingly, patients with advanced disease, that is, cases with IVC involvement, may benefit from surgery.^[5–7]^ In fact, pulmonary tumor embolism in patients with RCC may be considered an extension of vena caval tumors and not as distant metastasis; in this patient group, pulmonary tumor removal may result in a survival benefit.^[4]^ In that study, the authors also showed that concomitant nephrectomy with pulmonary embolectomy can be performed safely for patients with RCC with pulmonary tumor embolism, as no in-hospital mortality occurred in the 9 evaluated patients.^[4]^

Only a few retrospective studies with a small patient population have evaluated the clinical features and survival of adult patients with renal sarcoma. Moreira et al reported that surgery alone or in combination with radiotherapy was associated with improved cancer-specific survival of patients with non-metastatic as well as those with metastatic disease.^[3]^ In that study, among patients with metastatic disease, the 5-year survival rate was 40% for patients who underwent surgery compared with only 10% for those who did not undergo surgery; however, potential influence of selection bias should be taken into consideration. Moreover, the authors carefully concluded that resection could improve survival for both non-metastatic and metastatic renal sarcoma.^[3]^ In keeping with the above findings, Wang et al reported that resection was the only independent prognostic predictor for overall survival on multivariate analyses. The median overall survival was 8 months for the nonsurgical group versus 32 months for the surgical group.^[2]^ Thus, although we agree that more research is needed to establish the optimal treatment for primary renal sarcoma with massive pulmonary tumor embolism, which will still require some time for research, we believe that it is reasonable to perform surgery, as surgical approaches are effective for other renal neoplasms.

## 4 Conclusion

Surgeons usually expect patients with sarcoma (at any anatomical origin) with metastasis to have the worst prognosis. However, although renal sarcomas and RCCs are different, we believe that concomitant cytoreductive nephrectomy with pulmonary embolectomy combined with adjuvant chemotherapy may be effective for renal sarcoma with pulmonary tumor embolism, as it resulted in a good prognosis in the current patient.

## Author contributions

**Conceptualization:** Jun Ho Yang, Jong Woo Kim, Joung Hun Byun.

**Data curation:** Dong Hoon Kang, Jae Jun Jung.

**Investigation:** Seong Ho Moon.

**Methodology:** Chunwoo Lee, Dae Hyun Song.

**Project administration:** Joung Hun Byun.

**Supervision:** Jong Woo Kim.

**Writing – Original Draft:** Jun Ho Yang.

**Writing – Review & Editing:** Jun Ho Yang, Seong Ho Moon.
